# MicorRNA-195 links long non-coding RNA SEMA3B antisense RNA 1 (head to head) and cyclin D1 to regulate the proliferation of glioblastoma cells

**DOI:** 10.1080/21655979.2022.2052646

**Published:** 2022-03-31

**Authors:** Kaijun Liu, Yan Deng, Yongxia Yang, Hui Wang, Ping Zhou

**Affiliations:** a Department of Neurosurgery, Shiyan Taihe Hospital (Affiliated Taihe Hospital of Hubei University of Medicine), Shiyan City, PR. China; b Community Health Service Center of Shiyan Economic Development Zone, Shiyan City, PR. China

**Keywords:** Glioblastoma, SEMA3B-AS1, cyclin D1, miR-195, proliferation

## Abstract

Long non-coding RNA (lncRNA) SEMA3B antisense RNA 1 (head to head) (SEMA3B-AS1) is a recently identified tumor suppressor in gastric cancer. However, its role in glioblastoma (GBM) is unclear. This study was conducted to explore the role of SEMA3B-AS1 in GBM. In this study, the expression of SEMA3B-AS1, cyclin D1 and miR-195 were determined by RT-qPCR. Gene interactions were evaluated by dual-luciferase assay and overexpression experiments. BrdU assay was performed to monitor cell proliferation. We observed downregulation of SEMA3B-AS1 in GBM. The expression of SEMA3B-AS1 was inversely correlated with the expression of cyclin D1 but positively correlated with the expression of miR-195. In GBM cells, overexpression of SEMA3B-AS1 and miR-195 caused reduced expression levels of cyclin D1. MiR-195 inhibitor reduced the effects of overexpression of SEMA3B-AS1 on the expression of cyclin D1. Moreover, overexpression of SEMA3B-AS1 increased the expression levels of miR-195. Cell proliferation data showed that, SEMA3B-AS1 and miR-195 decreased cell proliferation, while overexpression of cyclin D1 increased GBM cell proliferation. In addition, miR-195 inhibitor inhibited the role of overexpression of SEMA3B-AS1 in cancer cell proliferation. Moreover, miR-195 interacted with cyclin D1, but not SEMA3B-AS1. Furthermore, SEMA3B-AS1 decreased the methylation of the promoter region of miR-195. Therefore, we concluded that miR-195 links lncRNA SEMA3B-AS1 and cyclin D1 to regulate the proliferation of GBM cells.

## Graphical abstract

**Figure:**
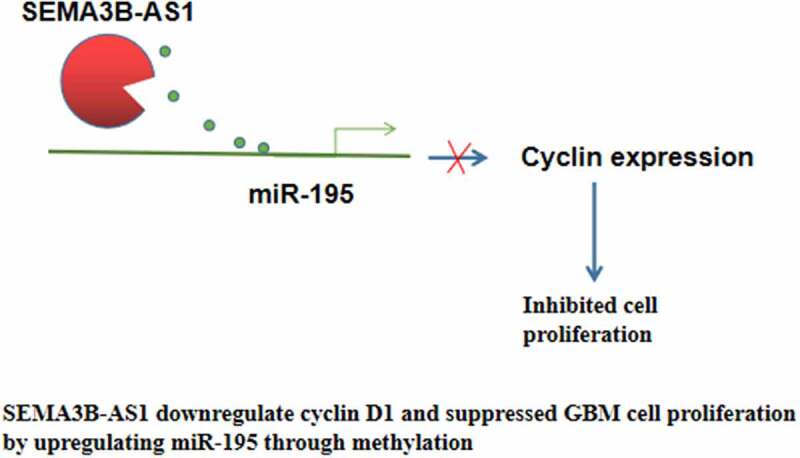

## Introduction

Glioma has four subtypes including oligodendrogliomas, mixed gliomas, ependymomas and astrocytic tumors. Glioblastoma (GBM) is a type of astrocytic tumor that accounts for about 80% of all brain cancer cases [1]. GBM is characterized by its high mortality rate. With treatment, GBM patients can only survive several months [2,3]. Even after active treatment, only 3% of GBM patients can survive more than 2 years and the median survival is only 15 months [2,3]. Based on histological findings, GBM belongs to grade IV gliomas with high proliferative rate and invasive and aggressive nature [4]. With the growth of aging population, the burden of GBM is predicted to be further increased [5].

Various factors affect the occurrence, development and progression of GBM, while genetic and epigenetic factors play central roles [6,7]. Long non-coding RNA (lncRNAs) participate in human cancers by regulating gene expression involved in cancer biology [^8–10^]. Cyclin D1 promotes cancer development by regulating cell cycle progression [11]. In cancer development, cyclin D1 can be regulated by both lncRNAs [12] and miRNAs [13], which lack protein-coding capacity but affect protein synthesis and the expression of other non-coding genes to regulate cancer progression. In effect, certain lncRNAs and miRNAs are promising targets for cancer therapy [12,13]. LncRNA SEMA3B antisense RNA 1 (head to head) (SEMA3B-AS1) has recently been shown to suppress gastric cancer [14]. Our preliminary microarray data showed that SEMA3B-AS1 was downregulated in GBM and inversely correlated with cyclin D1, which can be targeted by mi-R195. Moreover, our microarray data also revealed the positive correlation between SEMA3B-AS1 and miR-195. Therefore, we speculated that SEMA3B-AS1, miR-195 and cyclin D1 may interact with each other to participate in GBM. We then explored the involvement of SEMA3B-AS1 in GBM and analyzed its potential crosstalk with miR-195 and cyclin D1.

## Methods

### Research subjects

This study included 66 GBM patients (41 males and 25 females, age range 23 to 64 years old, mean age 44.3 ± 6.1 years old) who were selected from the 144 GBM patients admitted to Shiyan Taihe Hospital (Affiliated Taihe Hospital of Hubei University of Medicine) between June 2014 and September 2018. Patients with recurrent GBM, initiated therapy, other clinical disorders, family history or previous history of malignancies were excluded. All patients with glioblastoma underwent a CT scan, followed by MRI. Pathological diagnosis was performed using tumor tissues resected during surgery. All patients were Han Chinses. PTEN mutation was found in 10 cases, EGFR mutation was found in 12 cases, and TERT mutation was found in 11 cases. All patients were newly diagnosed GBM patients with no initiated therapy. Based on histological findings, all GBM patients were grade IV. This study was approved by the Ethics Committee of the aforementioned hospital. All patients were informed with the whole experimental protocol and the potential publication of the experiment data. All the 66 GBM patients signed the informed consent.

### Tissue collection and confirmation

Biopsy of tumor (GBM) as well as adjacent (within 2 cm around tumors) non-cancer tissues were collected from each patient. All specimens were confirmed through histopathological tests performed by 3 experienced pathologists. Tissue samples were stored in liquid nitrogen prior to the subsequent experiments.

### Cells and transient transfections

Human GBM cell lines U87 and U251 (ATCC® HTB14™, ATCC) were used. DMEM medium supplemented with 10% FBS and 100 U/ml penicillin/streptomycin was used to culture cells at 37°C with 5% CO_2_. SEMA3B-AS1 and cyclin D1 expression vectors (pcDNA3.1) were constructed by Sangon (Shanghai, China). SEMA3B-AS1 siRNA and NC siRNA were synthesized by Invitrogen (Shanghai, China). MiR-195 mimic and inhibitor as well as negative control (NC) were purchased from Sigma-Aldrich (USA). All transient cell transfections were performed using lipofectamine 2000 transfection reagent (Sigma-Aldrich, USA) to transfect 10 nM vectors, 30 nM miRNA, 30 nM siRNA or 30 nM inhibitor into 10^5^ cells. Transfection with empty pcDNA3.1 vector, negative control miRNA or inhibitor negative control was used as negative control (NC). Cells without transfection were used as the Control (C) cells.

### RT- qPCR

Ribozol was mixed with the ground tissues (ground in liquid nitrogen) or U87 cells (collected at 24 h after transfection) to isolate total RNAs. After digestion with DNase I, total RNA samples were reverse transcribed into cDNA using the SSRT III kit (Thermo Fisher Scientific, USA). With cDNA as template, qPCR was then performed to detect the expression of SEMA3B-AS1 and cyclin D1 with 18S rRNA as the endogenous control. miRNAs were also isolated from ground tissues (ground in liquid nitrogen) or U87 cells (collected at 24 h after transfection) using the microRNA Purification Kit (Norgen Biotek Corp, USA). Following the addition of poly (A) tail and reverse transcriptions, qPCRs were performed to analyze the expression of miR-545 with U6 as the endogenous control. PCR reactions were performed for 3 times and Ct values were analyzed using the 2^−ΔΔCT^ method [15]. Primer sequences were: 5’-CTCCAATATCTCAACCTCTC-3’ (forward) and 5’-GGGCACGTTCACCAGACTCA-3’ (reverse) for SEMA3B-AS1; 5’-GTAACCCGTTGAACCCCAT-3’ (forward) and 5’-CCATCCAATCGGTAGTAGC-3’ (reverse) for 18S rRNA; 5’-CCGTCCATGCGGAAGATC-3’ (forward) and 5’-ATGGCCAGCGGGAAGAC-3’ (reverse) for cyclin D1; 5’-CTTCGGCAGCACATATACTAAAAT-3’ (forward) and R:5ʹCGCTTCACGAATTTGCGTGTCA-3’ (reverse) for U6; 5’-TAGCAGCACAGAAATATTG-3’ (forward) and poly (T) (reverse) for miR-195.

### Dual-luciferase reporter assay

Dual-luciferase reporter assay was performed to detect luciferase activity of cells as previously described [16]. Luciferase vectors of SEMA3B-AS1 and cyclin D1 were prepared using pmirGLO Dual-Luciferase miRNA Target Expression Vector (Promega). Luciferase vector of SEMA3B-AS1 or cyclin D1 was co-transfected with miR-195 mimic into U87 cells. Cells were cultivated in 96-well plates and three replicate wells were set for each experiment. Renilla luciferase activity was measured at 48 h after transfection to normalize luciferase activities. Dual-Luciferase® Reporter Assay System (Promega) was used to complete all steps.

### BrdU incorporation assay

BrdU incorporation was used to reflect the proliferation of 6000 cells from each transfection group as previously described [17]. Three biological replicates of cells were treated with BrdU (10 µM) for 24 h. Cells were then fixed for 1 h and peroxidase-coupled anti-BrdU-antibody (Sigma–Aldrich) was used to incubate the cells for 60 min. Following PBS washing, cells were incubated with peroxidase substrate for 1 h. Finally, OD values at 450 nm were measured using the BioTek Epoch 2 Microplate Spectrophotometer.

### Methylation‐specific PCR

Genomic DNA was isolated from cells transfected with empty pcDNA3.1 vector or SEMA3B-AS1 expression vector using Wizard® Genomic DNA Purification Kit (Promega). Bisulfite modification was performed using EZ DNA Methylation‐Gold Kit (Zymo Research). Methyl primer express v1.0. software (Applied Biosystems) was used to design primers to amplify the promoter region (from position −125 to position – 1568) of miR-195.

### Western-blot analysis

U87 cells (10^5^ cells, collected at 24 h after transfection) were mixed with cell lysis buffer solution (Beyotime, Jiangsu, China) to extract total proteins. Total proteins were denatured and separated with 10% SDS-PAGE gel electrophoresis with 30 μg protein per lane, followed by transferring to PVDF membranes. After blocking with 5% non-fate milk, the membranes were incubated with primary antibodies GAPDH (1:2,000, ab37168, Abcam) and cyclin D1 (1:2,000, ab226977, Abcam) at 4°C overnight. The next day, IgG-HRP secondary antibody (1:1,000, MBS435036, Beijing Zhongshan Golden Bridge Biotechnology Co., Ltd) was used to incubate with the membranes at 22°C for 2 h. Finally, Enhanced chemiluminescence (Pierce SuperSignal, Thermo Scientific) was used for signal production.

### Statistical process

GraphPad Prism 6 was used for data analyses. All experiments were repeated for 3 times to calculate the mean values. Paired t test was used to compare paired tissues. ANOVA Tukey’s test was used to compare multiple groups. Linear regression was used to analyze correlations. Statistically significant level was *p*< 0.05.

## Results

### The expression of SEMA3B-AS1 in GBM

**Figure 1.**
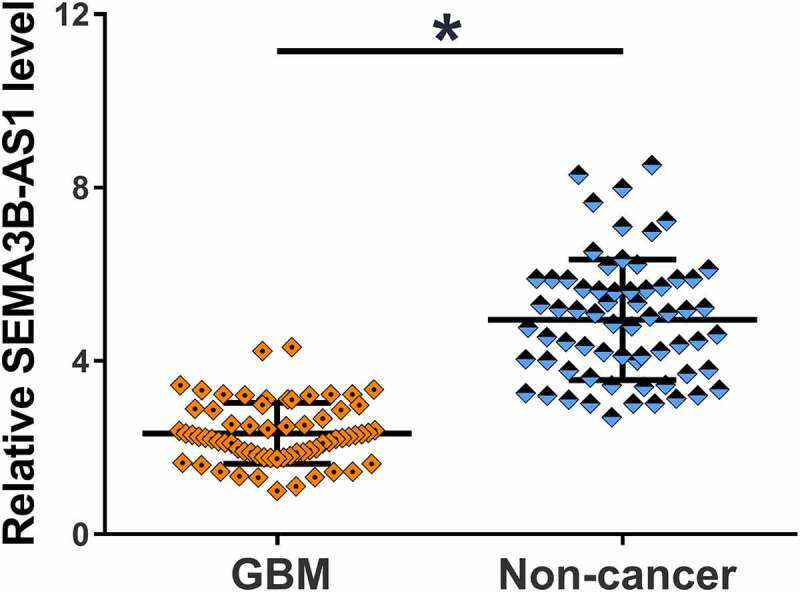
SEMA3B-AS1 was downregulated in GBM and affected by grades The expression of SEMA3B-AS1 in both GBM and non-tumor tissues were detected by RT-qPCR. PCR reactions were repeated for 3 times and mean values were compared by paired t test (*, *p* < 0.05).

The 66 patients were subjected to mutation analysis. According to NGS mutational analysis (Illumina MiSeq System) results, PTEN mutation was found in 10 cases, EGFR mutation was found in 12 cases and TERT mutation was found in 11 cases, and the rest 33 patients showed no mutation. To explore the potential involvement of SEMA3B-AS1 in GBM, the expression of SEMA3B-AS1 in GBM and non-cancer tissues from GBM patients was detected by performing RT-qPCR. In comparison to non-cancer tissues, GBM tissues exhibited significantly lower expression levels of SEMA3B-AS1 (Figure 1, *p* < 0.05). Therefore, the downregulation of SEMA3B-AS1 may participate in GBM.

### The expression of SEMA3B-AS1 in GBM tissues was significantly correlated with the expression of cyclin D1 and miR-195

**Figure 2.**
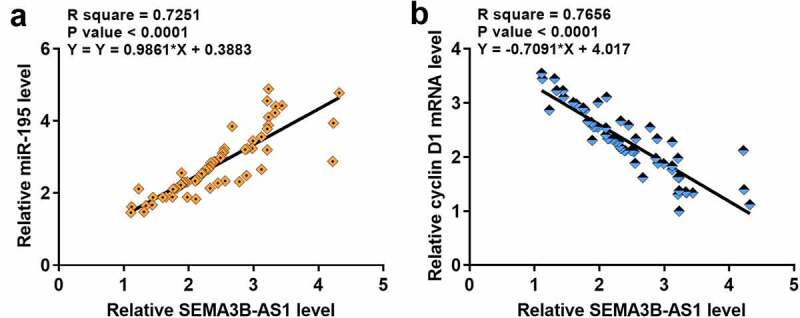
The expression of SEMA3B-AS1 in GBM tissues was significantly correlated with the expression of cyclin D1 and miR-195 The expression levels of cyclin D1 and miR-195 in GBM tissues were also measured by performing qPCR. Linear regression was used to analyze the correlations between the expression levels of SEMA3B-AS1 and cyclin D1 (a) or miR-195 (b) across GBM tissues.

The expression of cyclin D1 and miR-195 in paired GBM and non-cancer tissues from GBM patients was also detected by performing RT-qPCR. Compared to non-cancer tissues, cyclin D1 was significantly upregulated in GBM tissues (3.5-fold, data not shown), and miR-195 was significantly downregulated in GBM tissues (0.28-fold, data not shown). Correlations among SEMA3B-AS1, cyclin D1 and miR-195 were analyzed by performing linear regression. The expression of SEMA3B-AS1 was inversely and significantly correlated with the expression of cyclin D1 (Figure 2a), but positively and significantly correlated with the expression of miR-195 (Figure 2b). It is worth noting that our preliminary microarray data showed that SEMA3B-AS1 was downregulated in GBM (Supplemental Fig. 1A, *p* < 0.01) and inversely correlated with cyclin D1 (Supplemental Fig. 1B). Moreover, our preliminary microarray data also revealed the positive correlation between SEMA3B-AS1 and miR-195 (Supplemental Fig. 1B). Therefore, SEMA3B-AS1, cyclin D1 and miR-195 may interact with each other to participate in GBM.

### SEMA3B-AS1 regulated cyclin D1 through miR-195

**Figure 3.**
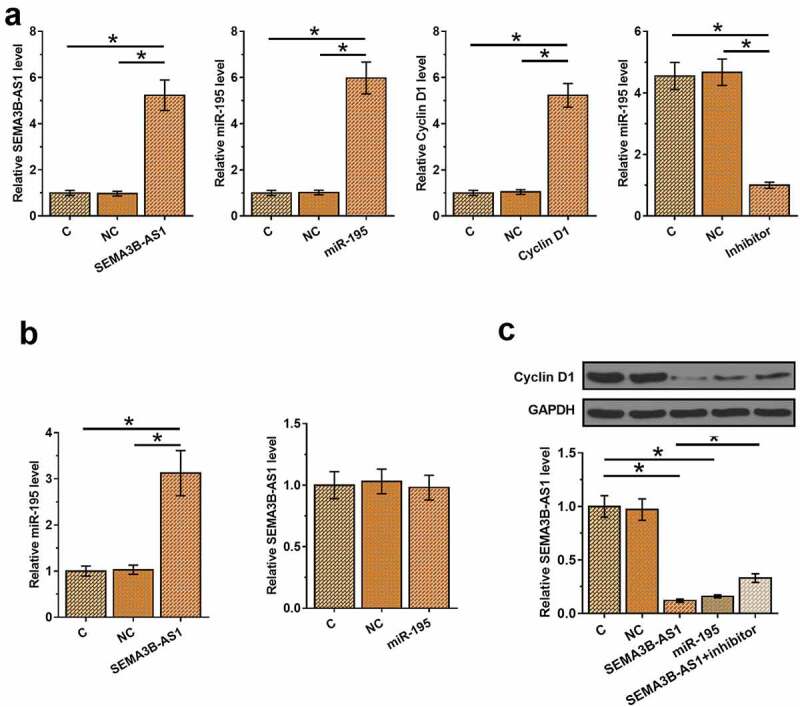
SEMA3B-AS1 regulated cyclin D1 through miR-195 U87 cells were transfected with SEMA3B-AS1 expression vector, miR-195 mimic, miR-195 inhibitor or cyclin D1 expression vector, and overexpression of SEMA3B-AS1, miR-195 and cyclin D1 or the downregulation of miR-195 was confirmed by qPCR at 24 h after transfection (a). The effects of overexpression of SEMA3B-AS1 and miR-145 on the expression of each other were also analyzed by performing qPCR (b). The roles of SEMA3B-AS1 and miR-195 in regulating cyclin D1 were analyzed by qPCR and western blot analysis (c). All experiments were repeated 3 times and mean values were compared. C, cells without transfection; NC, cells transfected with NC miRNA, NC inhibitor or empty vector. (*, *p* < 0.05).

To explore the relationship among SEMA3B-AS1, cyclin D1 and miR-195, SEMA3B-AS1 or cyclin D1 expression vector, miR-195 mimic or miR-195 inhibitor was transfected into U87 cells. Compared to C and NC, the expression of SEMA3B-AS1, miR-195 and cyclin D1 were significantly altered at 24 h after transfection (Figure 3a, *p* < 0.05), indicating successful transfections. Moreover, compared to C and NC, overexpression of SEMA3B-AS1 also increased the expression levels of miR-195, while overexpression of miR-195 did not affect the expression of SEMA3B-AS1 (Figure 3b, *p* < 0.05). Overexpression of SEMA3B-AS1 and miR-195 reduced the expression levels of cyclin D1, and miR-195 inhibitor attenuated the effects of overexpression of SEMA3B-AS1 on cyclin D1 expression (Figure 3c, *p* < 0.05). The interaction between SEMA3B-AS1 and miR-195, and the interaction between miR-195 and cyclin D1 was explored by performing Dual-luciferase reporter assay. It was observed that miR-195 did not alter the luciferase activity of SEMA3B-AS1 vector (Supplemental Fig. 2A), but decreased the luciferase activity of SEMA3B-AS1 vector (Supplemental Fig. 2B, *p* < 0.01).

### SEMA3B-AS1 inhibited U87 cell proliferation through cyclin D1 and miR-195 in both U87 and U251 cells

**Figure 4.**
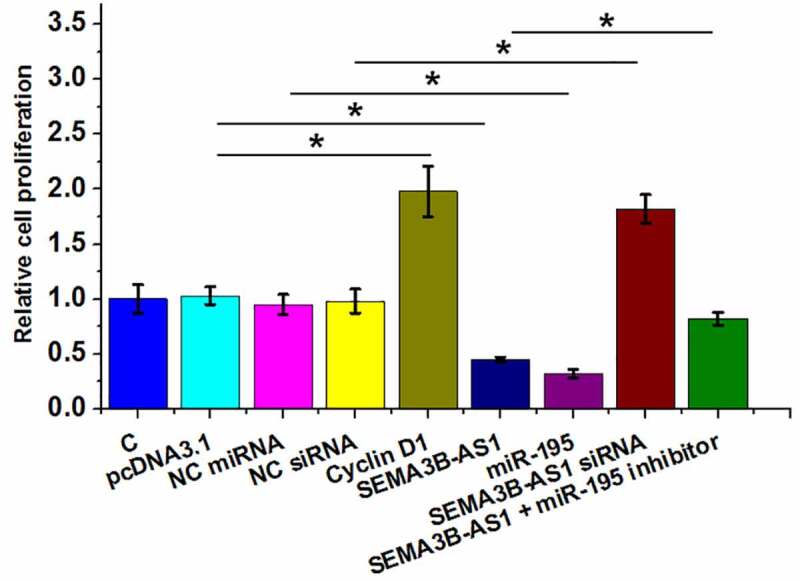
SEMA3B-AS1 inhibited U87 cell proliferation through cyclin D1 and miR-195 The roles of EMA3B-AS1, cyclin D1 and miR-195 in regulating the proliferation of U87 cells were analyzed by cell proliferation (BrdU) assay. All experiments were repeated 3 times and mean values were presented and compared. (*, *p* < 0.05).

Cell proliferation contributes to the progression of GBM. To this end, the role of SEMA3B-AS1, cyclin D1 and miR-195 in regulating U87 cell proliferation was analyzed by BrdU assay. The results showed that overexpression of SEMA3B-AS1 and miR-195 decreased cell proliferation, while silencing of SEMA3B-AS1 and overexpression of cyclin D1 increased of the proliferation rate of GBM cells. In addition, miR-195 inhibitor attenuated the effects of overexpression of SEMA3B-AS1 on cancer cell proliferation (Figure 4). Therefore, SEMA3B-AS1 my inhibit GBM cell proliferation by downregulating cyclin D1 through miR-195. The same experiment was also performed on U251 cells and similar results were obtained (Supplemental Fig. 3).

### SEMA3B-AS1 increased the methylation of miR-195 promoter

**Figure 5.**
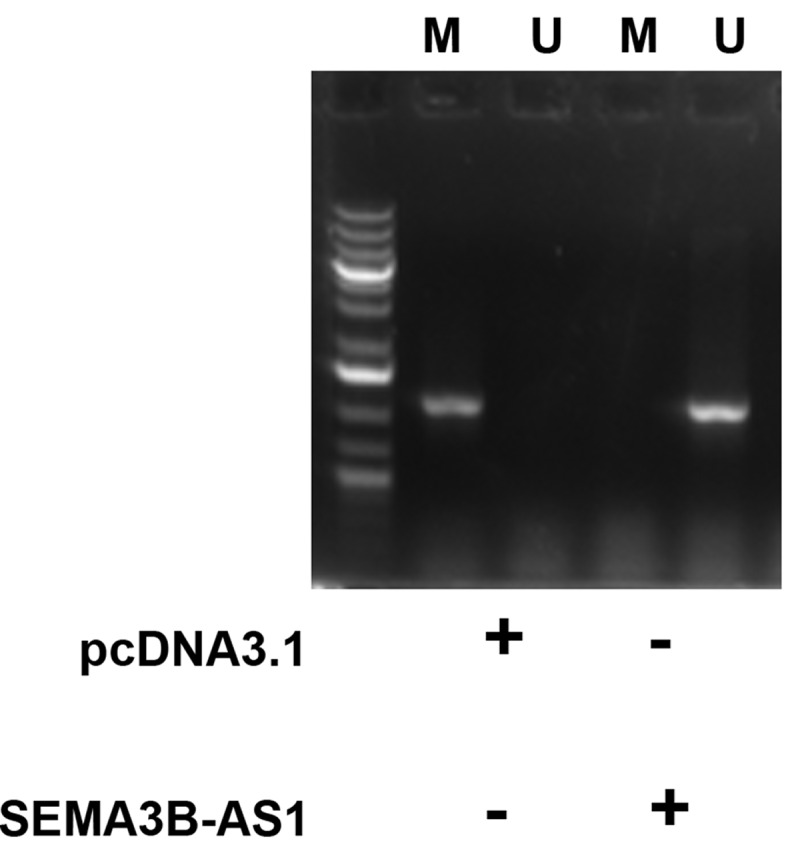
SEMA3B-AS1 increased the methylation of miR-195 promoter U87 cells were transfected with empty pcDNA3.1 vector or EMA3B-AS1 vector, followed by performing MSP to detect the methylated (m) and un-methylated (u) promoter of miR-195 gene.

MSP was performed to analyze the role of SEMA3B-AS1 in regulating the methylation of miR-195 promoter. Compared to cells transfected with empty pcDNA3.1 vector, cells transfected with SEMA3B-AS1 vector showed decreased methylation of miR-195 promoter (Figure 5). Therefore, SEMA3B-AS1 may upregulate miR-195 by reducing the methylation of its promoter.

## Discussion

Treatment of GBM always needs novel therapeutic targets. In the present study we investigated the role of SEMA3B-AS1 in GBM and found that SEMA3B-AS1 is a tumor suppressor in GBM. In addition, we also characterized the involvement of the SEMA3B-AS1/miR-195/cyclin D1 axis in the proliferation of GBM cells.

Inhibition of cancer cell proliferation is always essential for clinical treatment of cancer. D cyclins play a pivotal role in cell cycle progression by forming complex with CDK4 or CDK6 to phosphorylate the retinoblastoma and drive the transition from G1 to S phase, thereby promoting cell proliferation [18]. Cyclin D1 is member of D cyclins that is usually upregulated in cancers including GBM [19,20]. A recent study reported that cyclin D1 is a direct target of miR-195 in glioma [13]. The present study also observed the downregulated expression of cyclin D1 in GBM cells after overexpression of miR-195.

LncRNAs may have similar functionalities in different types of cancer. For example, lncRNA HOTAIR shows similar functions in regulating cancer cell behaviors in different types of cancer [21]. SEMA3B-AS1 was downregulated in gastric cardia adenocarcinoma and overexpression of SEMA3B-AS1 resulted in inhibited cancer cell proliferation. Similarly, we also found the downregulation of SEMA3B-AS1 in GBM and the suppressed proliferation of GBM cells after overexpression of SEMA3B-AS1. However, our study did not detect the expression of SEMA3B-AS1 in patients’ plasma (data not shown). Circulating lncRNAs released into blood from the site of synthesis have been widely used to predict cancer development [22], while SEMA3B-AS1 may be not a good option possibly due to its low level in blood.

The interactions between lncRNAs and miRNAs have been extensively investigated in previous studies [23]. LncRNAs may serve as the sponge of miRNAs to inhibit their roles [24]. However, the mechanism of the upregulation of miRNAs by lncRNAs is largely unknown. The present study showed that SEMA3B-AS1 was an upstream positive regulator of miR-195, and the upregulation of miR-195 by SEMA3B-AS1 is involved in the regulation of cyclin D1 and GBM cell proliferation. Interestingly, we observed that SEMA3B-AS1 can decrease the methylation of miR-195 promoter in GBM cells. Therefore, SEMA3B-AS1 may upregulate miR-195 in GBM by reducing the methylation of its promoter. Our study characterized a novel SEMA3B-AS1/miR-195/cyclin D1 axis involved in GBM, while the upregulation of miR-195 by SEMA3B-AS1 still needs to be further investigated. A recent study reported that lncRNA RUNX1-IT1 can also sponge miR-195 to upregulate cyclin D1 in glioblastoma, thereby promoting cell proliferation [25]. Therefore, miR-195 in GBM may be sponged by multiple lncRNAs.

## Conclusion

In conclusion, SEMA3B-AS1 is downregulated in GBM and overexpression of SEMA3B-AS1 can downregulate cyclin D1 and suppress GBM cell proliferation possibly through miR-195. With the increased understanding of the molecular mechanism of GBM [26,27], lncRNAs are expected to be targeted to treat GBM.

## Supplementary Material

Supplemental Material

## Data Availability

The analyzed data sets generated during the study are available from the corresponding author on reasonable request.

## Abbreviations

Glioblastoma (GBM) long non-coding RNAs (lncRNAs, > 200 nt)
