# Supplementary figures and images for: MicorRNA-195 links long non-coding RNA SEMA3B antisense RNA 1 (head to head) and cyclin D1 to regulate the proliferation of glioblastoma cells
Source: Bioengineered. 2022 Mar 31;13(4):8798–805. doi: 10.1080/21655979.2022.2052646 (PMC9161951; doi:10.1080/21655979.2022.2052646)

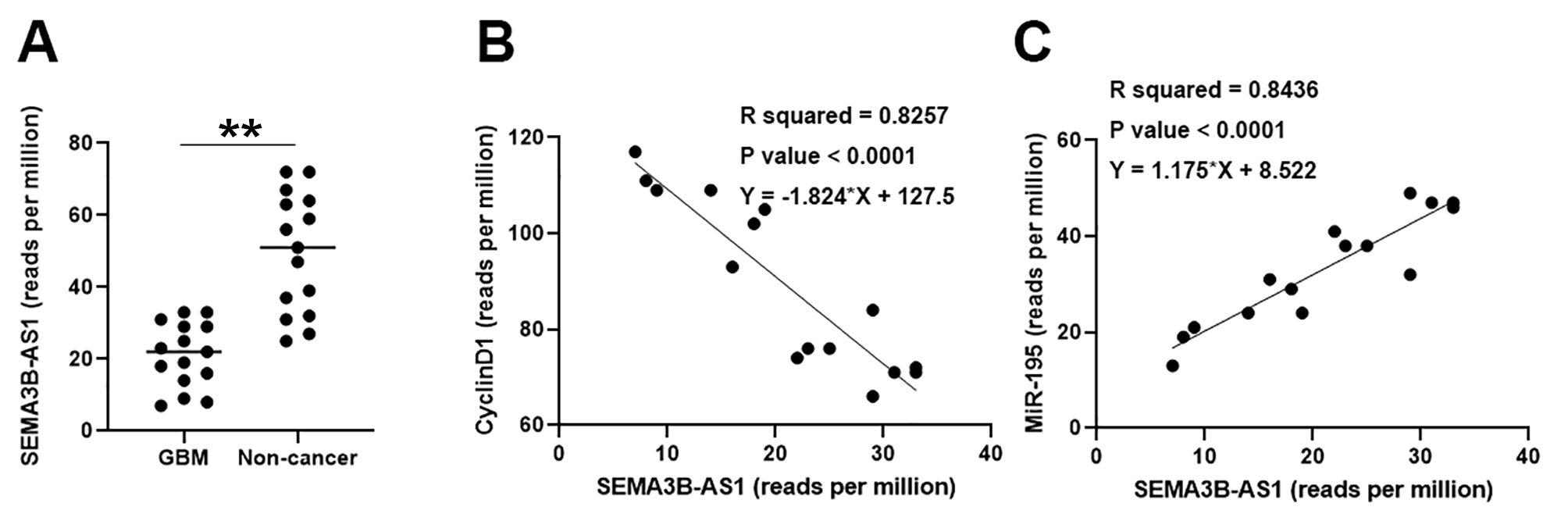

Supplement: Supplemental Material [file KBIE_A_2052646_SM2032.zip › Supplemental Figure1.tif]

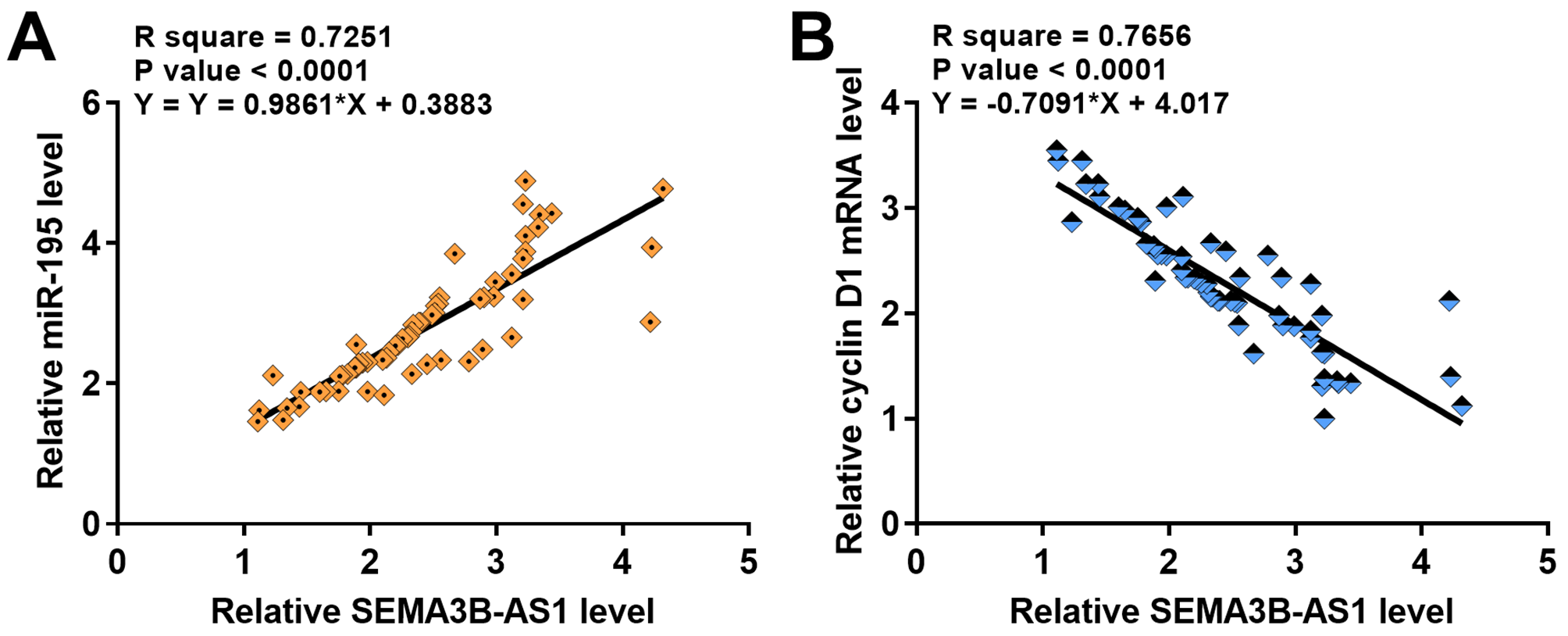

Supplement: Supplemental Material [file KBIE_A_2052646_SM2032.zip › Supplemental Figure2.tif]

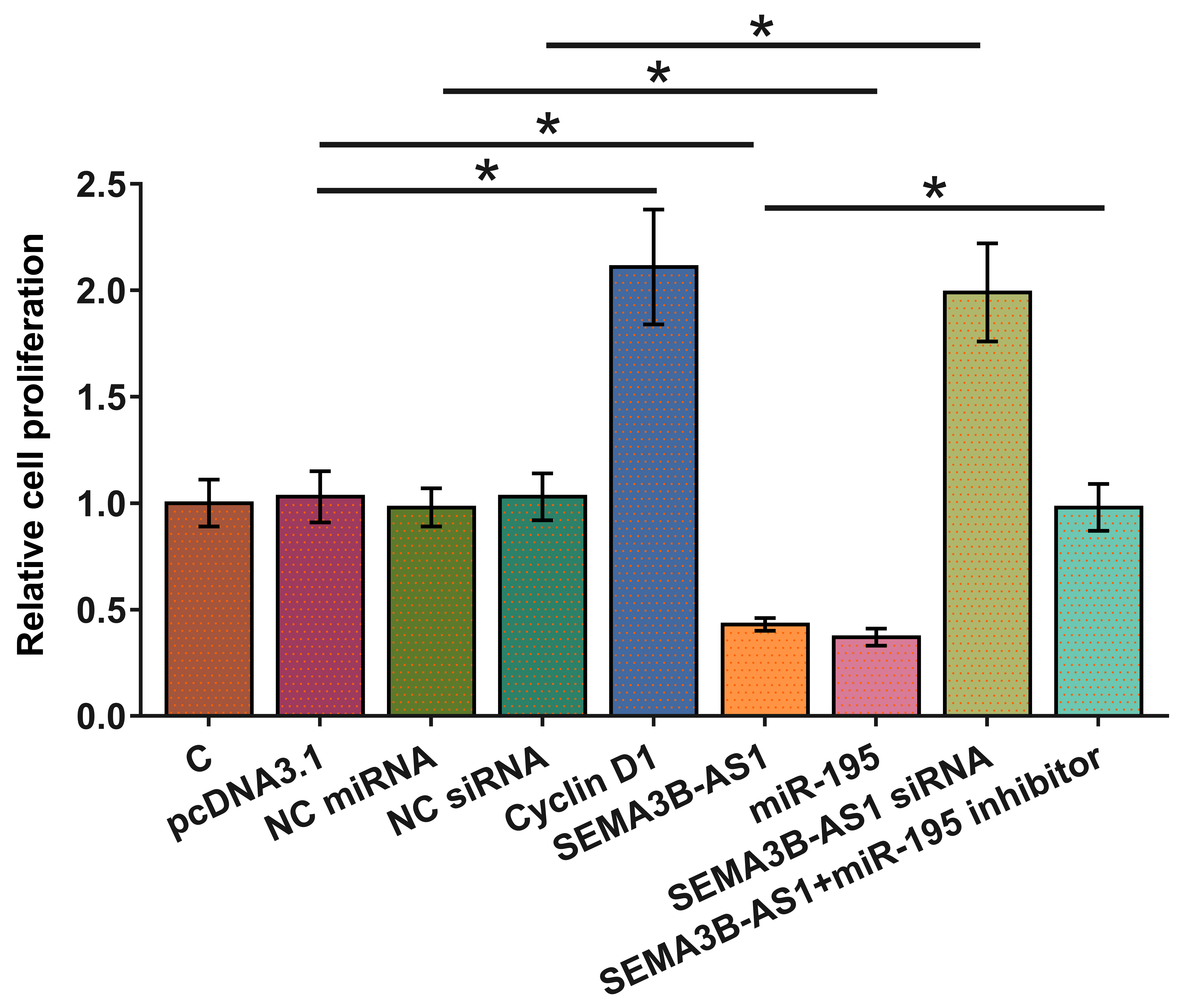

Supplement: Supplemental Material [file KBIE_A_2052646_SM2032.zip › Supplemental Figure3.tif]
